# Antibiotic Pigment from Desert Soil Actinomycetes; Biological Activity, Purification and Chemical Screening

**DOI:** 10.4103/0250-474X.58174

**Published:** 2009

**Authors:** L. Selvameenal, M. Radhakrishnan, R. Balagurunathan

**Affiliations:** Department of Microbiology, Sri Sankara Arts and Science College, Kanchipuram-631 561, India; 1 Department of Microbiology, Periyar University, Salem-636 011, India

**Keywords:** Antimicrobial pigment, drug-resistant pathogens, bioautography, chemical screening

## Abstract

An actinomycete strain, *Streptomyces hygroscopicus* subsp. ossamyceticus (strain D10) was isolated from Thar Desert soil, Rajasthan during the year 2006 and found to produce a yellow color pigment with antibiotic activity. Crude pigment was produced from strain D10 by solid state fermentation using wheat bran medium followed by extraction with ethyl acetate. The antimicrobial activity of the crude pigment was evaluated against drug resistant pathogens such as methicillin-resistant *Staphylococcus aureus,* vancomycin-resistant *Staphylococcus aureus,* extended spectrum β-lactamase producing cultures of *Escherichia coli, Pseudomonas aeruginosa* and *Klebsiella* sp. About 420 mg of crude pigment was produced per 10 g of wheat bran medium. In the disc diffusion method the crude ethyl acetate extract showed a minimum of 10 mm inhibition against *Klebsiella* sp. and maximum of 19 mm of inhibition against *Escherichia coli.* The crude pigment was partially purified using thin layer chromatography with the solvent system chloroform:methanol (30:70) and the Rf value was calculated as 0.768. Antimicrobial activity of the partially purified compound from thin layer chromatography was determined using the bioautography method. The purified pigment showed minimum of 15 mm inhibition against Klebsiella sp. and a maximum of 23 mm of inhibition against vancomycin-resistant *Staphylococcus aureus* in the disc diffusion method. Based on the results of chemical screening, the pigment was tentatively identified as group of sugar containing molecules.

Infectious diseases are the leading cause of death world wide accounting for 13.3 billion deaths, which constitutes about 25% of all deaths. At present, resistance to the drugs used in the treatment of many infectious diseases is increasing, while microbial infections are being found to be responsible for more life threatening diseases than previously thought. The reasons for the increase in incidence of infectious diseases are not fully understood. One such reason is the emergence of multidrug resistant pathogens[1]. Among the various drug resistant pathogens, methicillin-resistant *Staphylococcus aureus* (MRSA), vancomycin-resistant *Staphylococcus aureus* (VRSA), extended spectrum β-lactamases (ESBL) producing bacteria such as *E. coli, Klebsiella* sp. and *Pseudomonas aeruginosa* and multi drug resistant *Mycobacterium tuberculosis* (MDR-MTB) are of major concern.

The demand for new antibiotics continues to grow due to the rapid emergence of antibiotic resistant pathogens causing life threatening infections in spite of considerable progress in the fields of chemical synthesis and engineered biosynthesis of antimicrobial compounds. This changing pattern of diseases and the emergence of resistant bacterial strains to currently used antibiotics continuously puts demand on the drug discovery scientists to search for novel antibiotics[2].

Actinomycetes perform significant biogeochemical roles in terrestrial soils and are highly valued for their unparalleled ability to produce biologically-active secondary metabolites. Totally 22,500 bioactive secondary metabolites have been reported, out of which 16,500 compounds show antibiotic activities. Out of the 22,500 total bioactive secondary metabolites, 10,100 (45%) are reported to be produced by actinomycetes in which 7630 from streptomycetes and 2470 from rare actinomycetes. A search of recent literature revealed that atleast 4607 patents have been issued on actinomycete related products and processes[3].

In the past two decades, however, there has been a decline in the discovery of new lead compounds from common soil-derived actinomycetes, as culture extracts usually yield unacceptably high number of previously described metabolites. The immense biotechnological ability of actinomycetes had led to exhaustive surveys of cultivars from normal terrestrial habitats and an associated increase in the number of known compounds being rediscovered due to a high rate of redundancy in the strains isolated. For this reason, searching the less or unexploited ecosystems for actinomycetes might lead to the discovery of novel bioactive compounds including those that can act against drug-resistant pathogens[4].

Recently scientists started searching various extreme ecosystems such as deep sea[5], forest, mountains[6,7] and deserts for the discovery of novel actinomycetes and antibiotics. In an attempt to search unexploited ecosystems, an actinomycete strain *Streptomyces hygroscopicus subsp. ossamyceticus* (strain D10) was isolated from desert soil, Rajasthan during the year 2006. An extracellular yellow pigment produced by this strain showed good antibacterial and antimycobacterial activity[8]. As a part of further evaluation of biological activity, purification and chemical screening of antimicrobial pigment from strain D10, the present work is undertaken.

## MATERIALS AND METHODS

### Actinomycetes strain:

**Fig. 1:**
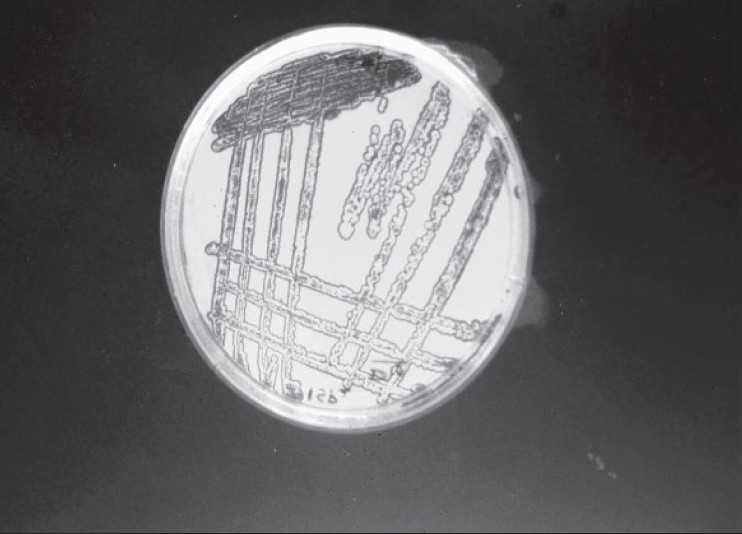
Growth of strain D10 on ISP2 agar Growth of actinomycete strain D10 on yeast extract malt extract agar

**Fig. 2:**
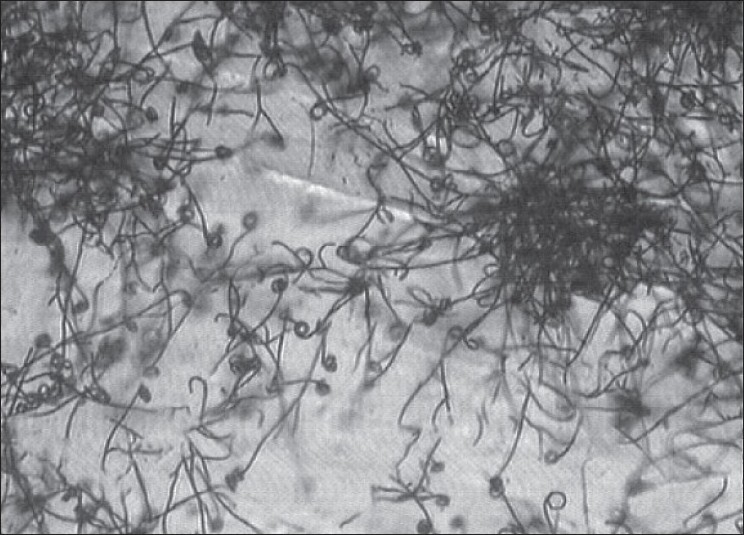
Micromophology of strain D10 Micromorphology of actinomycete strain D10 under bright field microscope (400 x)

**Fig. 3:**
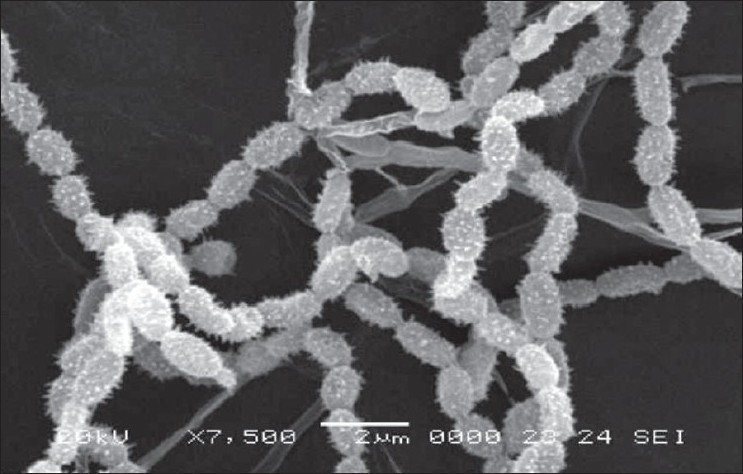
SEM of actinomycete strain D10 Mcromorphology of actinomycete strain D10 under scanning electron microscope (7500 x)

The actinomycete strain used in this study was isolated from Thar Desert soil during 2006 and found to produce a yellow color pigment (figs 1–3). The strain was cultivated on yeast extract malt extract agar for 7 d at 28° and preserved in refrigerator until further studies. During the preliminary screening the pigment producing actinomycete strain D10 was studied for its antibacterial activity by cross streak method against multidrug resistant pathogens such as MRSA, VRSA and ESBL producing strains of *Escherichia coli, Klebsiella* sp., and *Pseudomonas aeruginosa.* In this study, the strain D10 showed good antibiotic activity against all the bacterial pathogens tested except *Pseudomonas aeruginosa*[8].

### Test pathogens:

All the clinical bacterial isolates and their antibacterial susceptibility data were obtained from the Department of Microbiology, Sri Sankara Arts and Science College, Kanchipuram. The MRSA strain was resistant to penicillin, methicillin and vancomycin; and the VRSA strain was resistant to penicillin, methicillin and vancomycin. *E. coli, Klebsiella* sp. and *Pseudomonas aeruginosa* were resistant to antibiotics such as amoxicillin, ceftriaxone, cephotaxime and azetreonam.

### Production of crude pigment by solid state fermentation:

Mayuran *et al.*[8] proved that solid state fermentation was better for the production of pigment by the strain D10 when compared to submerged fermentation. Hence in this present study, solid state fermentation was adopted for the production of crude pigment. For the preparation of inoculum, the actinomycete strain D10 was streaked on the yeast extract malt extract agar (ISP2 medium) plates and incubated at 28° for 5 d. The spores were scrapped from the plate and inoculated into 50 ml of yeast extract malt extract broth (ISP2) medium and incubated in rotary shaker for 48 h with 120 rpm at 28°. About 10 g of wheat bran was added into 250 ml conical flask with 5 ml of distilled water and sterilized. Then 10% of inoculum was added into conical flask containing sterile wheat bran. The flasks were incubated at 28° for 10 d.

### Extraction of crude pigment:

After incubation, the fermented biomass was mixed with 50 ml of ethyl acetate and macerated by using mortar and pestle. As ethyl acetate was standardized as the best solvent to extract the pigment, the crude pigment was collected and concentrated by evaporation[8]. Quantity of crude pigment was measured by adding the pigment into the dried 25 ml preweighed beaker. After evaporation of the solvent, the weight of crude pigment was measured and stored in sterile vials[9].

### Biological activity of crude pigment

The crude pigment was screened for biological activity against drug resistant pathogens MRSA, VRSA and ESBL cultures of *E. coli, Klebsiella* sp*., Pseudomonas aeruginosa* by disc diffusion method. About 0.25 mg of crude ethyl acetate extract (pigment) was impregnated with sterile filter paper disc. Then 18 h old broth cultures of test bacterial pathogens were inoculated by making a lawn on nutrient agar by using sterile cotton swab. The crude pigment impregnated discs were placed over the test organisms on nutrient agar. The diameter of the inhibition zone was measured after 24 h of incubation at 37°[10].

### Purification of crude pigment by thin layer chromatography:

The crude pigment was purified by using thin layer chromatography[10]. Commercially available Silica gel coated chromatography sheets (50×20 cm size) were used. To find out the best solvent system to separate the crude compound, the solvents such as methanol, chloroform, acetic acid, *n*-butanol, *n*-hexane and water were used in different proportions. *n*-hexane:chloroform (40:60, 50:50, 60:40), *n*-butanol:acetic acid:water (70:20:10 and 60:30:10) and chloroform:methanol (60:40, 50:50, 70:30, 30:70, 80:20 and 20:80) were used.

The crude pigment was dissolved in 200 μl of ethyl acetate. With the help of capillary tube, the sample was spotted at the bottom of silica gel coated sheet and then it was placed in the developing beaker containing mobile phase, covered with the watch glass in order to prevent the evaporation of the solvents. The solvent was allowed to run till it reaches about half a centimeter below the top of the plate. After running, the sheet was kept at room temperature for the complete drying of the plate. Then the sheet was kept in closed iodine chamber to visualize the separated compound as clear sports. Among all solvent systems used, chloroform: methanol (30:70) showed good separation. Rf value of the spot separated on the TLC plate was determined. Rf value = movement of solute from the origin/movement of solvent from the origin

### Detection of active compound by bioautography:

The bioautography method described by Rahalison *et al.*[11] followed for the detection of active compound separated in TLC. Chromatogram developed as described above was placed in a sterile bioassay Petri dish and overlaid with 10 ml molten nutrient agar seeded with 0.2 ml of each of the test organisms such as MRSA, VRSA and ESBL cultures of *E. coli, Klebsiella* sp*., Pseudomonas aeruginosa* and incubated overnight at 37° for 24 h.

### Preparative TLC:

TLC plate was prepared by spreading the slurry of silica gel evenly on the plate. The plate was activated at 100° for 15 min. Crude pigment of D10 spotted on the plate as a single line or streak and the chromatogram was performed with the solvent system chloroform:methanol (30:70). After drying, the pigment spot was scrapped, mixed with ethyl acetate and centrifuged at 3000 rpm for 15 min. Supernatant was collected in a preweighed vial and kept for evaporation. The partially purified pigment obtained from preparative TLC was tested for antimicrobial activity against the drug resistant bacterial pathogens by disc diffusion method as described earlier.

### Chemical screening:

Chemical screening of partially purified compound was performed to determine the functional group present in the bioactive compound as recommended by Fiedler[12]. Commercially available readymade TLC sheets (Silica gel 60- F254 nm) spotted with crude pigment. Chloroform:methanol (30:70) was used as a solvent system. Spots were obtained after the solvent gets eluted. After drying, the sheets were sprayed with the following reagents. The sheets were kept in hot air oven at 120° to observe the colour change.

### Spraying reagents:

One gram of dimethylaminobenzaladehyde was mixed with 25 ml of 36% HCl and 75 ml of methanol. The stained sheets were heated to about 120° for a few minutes till maximal colouration. This reagent was specific for primary amines. Reagent A consisted of 0.5 g of blue tetrazolium mixed with 100 ml of methanol. Reagent B was prepared by mixing 24 g of sodium hydroxide in 50 ml water with 50 ml methanol. Reagent A and B were mixed 1:1 before use. The stained sheets were heated to about 120° for few minutes till maximal colouration. Blue or violet colour zone formation or a light background will indicates the positive result. This reagent was specific for steroids and reducing compounds.

One gram vanillin was mixed with 100 ml concentrated sulphuric acid. The stained sheets were heated to about 120° for few minutes till maximal colouration. Coloured zones produced on a pale background were the indication of positive result. This reagent was specific for higher alcohols, phenols, steroids.

Reagent 1 consisting of 0.2 g naphthalene-1,3-diol in 100 ml ethanol was mixed 1:1 with reagent 2, which is 20% Sulphuric acid, just before use. The stained sheets were heated to about 120° for few minutes till maximal colouration. Red, blue, green, violet, brown, orange or yellow coloured zone formation were the indication of positive results. This reagent was relatively specific for sugars.

## RESULTS

### Production and extraction of pigment by solid state fermentation:

Strain D10 showed good mycelial growth on wheat bran medium after fermentation. The color of the substrate was changed to dark yellow with greyish mycelial growth. Quantity of crude pigment extracted by using wheat bran as a substrate was found to be 420 mg/10 g of wheat bran.

### Separation of crude pigment:

The solvent system chloroform:methanol (30:70) was found to have good separation with single spot when compared to all the solvent systems used for TLC. The Rf value of the spot was found to be 0.768.

### Antimicrobial activity of crude pigment:

The crude pigment showed activity against MRSA, VRSA and ESBL cultures of *E. coli* and *Klebsiella* sp. but not against *Pseudomonas aeruginosa*. Antimicrobial activities of crude pigment against drug resistant pathogens were given in the Table 1. Antimicrobial activity of the partially purified compound from TLC was tested by bioautography method. The inhibition zone on chromatogram was observed and the results were given in Table 2.

Preparative TLC was performed to get more amount of partially purified compound using chloroform:methanol (30:70) as a solvent. The spot was obtained after the completion of separation. After drying, the spot was scrapped, mixed with ethyl acetate, centrifuged at 3000 rpm for 15 min. Supernatant was collected and kept for evaporation. The quantity of partially purified compound was found to be 30 mg. The antimicrobial activity of the partially purified pigment was given in Table 3. When compared to crude pigment, partially purified compound showed increased zone of inhibition against the tested bacterial pathogens.

**TABLE 1:** ANTIMICROBIAL ACTIVITY OF CRUDE PIGMENT

Test organisms	Inhibition zone (diameter in millimeter)
MRSA	15
VRSA	17
*Escherichia coli*	19
*Klebsiella sp.*	10
*Pseudomonas aeruginosa*	--

negative reaction for zone of inhibition

**TABLE 2:** ANTIMICROBIAL ACTIVITY OF PIGMENT SEPARATED IN BIOAUTOGRAPHY

Test organisms	Inhibition zone (diameter in millimeter)
MRSA	15
VRSA	20
*Escherichia coli*	15
*Klebsiella sp.*	10
*Pseudomonas aeruginosa*	--

negative reaction for zone of inhibition

**TABLE 3:** ANTIMICROBIAL ACTIVITY OF PARTIALLY PURIFIED PIGMENT FROM PREPARATIVE TLC

Test organisms	Inhibition zone (diameter in millimeter)
MRSA	20
VRSA	23
*Escherichia coli*	20
*Klebsiella sp.*	15
*Pseudomonas aeruginosa*	--

negative reaction for zone of inhibition

### Chemical screening:

In chemical screening, among the different spraying reagents used, red colouration was observed while using naphthoresorcin-sulphuric acid. Based on the observation, the functional group of the active pigment was tentatively identified as sugar molecules.

## DISCUSSION

Antibiotic resistant pathogens pose an enormous threat to the treatment of a wide range of serious infections. To prevent this exponential emergence, a periodic replacement of the existing antibiotic is necessary[13]. Currently, the greatest cause of concern is infection caused by methicillin and vancomycin resistant strains of *Staphylococcus aureus*, ESBL strains of *E. coli, Klebsiella* *sp.* and *Pseudomonas aeruginosa.* The development of novel drugs against drug resistant pathogen is the need of the hour.

From the ten thousands of known microbial metabolites about 150-160 (0.2-0.3%) compounds were practically proved as successful lead compounds. The value of actinomycetes to society in terms of providing useful drugs, and to the pharmaceutical industry for revenue generating discovery platform is indisputable. Actinomycete products such as antibiotics like streptomycin and novobiocin are firmly cemented these chemically prolific bacteria in the centre stage of natural products drug discovery research[14].

Some of the antibiotics produced from *Streptomyces hygroscopicus* are carriomycin, holomycin etc. which showed activity against normal bacterial pathogens. It has long been known that some of the actinomycete strains of the same species could generate different antibiotics, where as some other strains belonging to different species generated the same antibiotics[15]. In this present study, the pigment producing actinomycete strain D10 (*Streptomyces hygroscopicus*) showed antibacterial activity against the drug resistant pathogens such as MRSA, VRSA and ESBL strains. The production of antibiotics by actinomycetes therefore, may not be species specific but rather strain specific[16].

Solid state fermentation holds an important potential for the production of secondary metabolites especially from filamentous organisms such as fungi and actinomycetes[17]. In this present study crude pigment was produced from strain D10 in high quantity (420 mg/10 g of wheat bran substrate). These results suggested that solid state fermentation using wheat bran medium is the best method for the production of antimicrobial pigment from *Streptomyces hygroscopicus* (D10).

Sathi *et al.*[18] isolated yellowish antibiotic pigment 4-hydroxynitrobenzene from *Streptomyces* species. The yellow pigment was extracted in chloroform and tested against *Bacillus subtilis* and *Shigella shiga*. But in this present study the crude yellowish antibiotic pigment showed good separation with one spot of Rf value 0.768. The result obtained in the present study was different from the yellowish antibiotic pigment reported from *Streptomyces species* by Sathi *et al*[18].

Bioautography allows localizing antimicrobial activity of an extract on the chromatogram, it supports a quick search for new antimicrobial agent through bioassay guided isolation. This method avoids the need of previous purification of the substance, reducing the cost of the initial screening[19]. In this present study activity of purified compound was detected in TLC sheet itself by agar overlay bioautography. In bioautography, the separated spot showed activity against the drug resistant pathogens tested. Based on the results of bioautography, the crude compound was purified by preparative TLC and the purified compound also showed good activity in disc diffusion studies (15-23 mm zone of inhibition) against drug resistant pathogens.

The strategy for discovery of new lead structures was described by Zahner[20] who developed a non target screening method based on thin layer chromatography combined with different spray reagents for the detection of new secondary metabolites. This so called chemical screening led to the isolation of a variety of new bioactive compounds[12]. In the present study the crude compound purified in TLC was tested for chemical screening by using different spray reagents. The active compound was identified as sugar molecules.

The present study concludes that the yellowish antibiotic pigment produced by the desert soil actinomycete *Streptomyces hygroscopicus subsp. ossamyceticus* (D10) was suspected as a novel one, since it is isolated from extreme ecosystem of Thar Desert. It's also a potential antibiotic for the treatment of diseases caused by drug resistant bacterial pathogens including *Mycobacterium tuberculosis*. In addition, the yellow pigment can also be used as a food colorant and preservatives, if the toxicity tests are found negative. Microbial pigments such as carotenoids, melanins and flavins are used in food industries[21]. Studies like HPLC purification, chemical characterization and structure elucidation is in progress to prove its potential.

## References

[1] Cassell GH, Mekalanos J (2001). Development of antimicrobial agents in the era of new and reemerging infectious diseases and increasing antibiotic resistance. J Am Med Assn.

[2] Baltz RH (2007). Antimicrobials from actinomycetes: Back to the Future. Microbe.

[3] Berdy J (2005). Bioactive microbial metabolites. A personal view. J Antibiot.

[4] Mincer TJ, Jenson PR, Kauffman CA, Fenical W (2002). Widespread and persistent populations of a major new marine actinomycete taxon in the ocean sediments. Appl Environ Microbiol.

[5] Jenson PR, Mincer TJ, Williams PG, Fenical W (2005). Marine actinomycete discovery and natural product discovery. Antonie van Leeuwenhoek.

[6] Valan AM, Duraipandian V, Agastian P, Ignacimuthu (2008). Antimicrobial activity of Streptomyces spp. ERI-26 recovered from Western Ghats of Tamil Nadu. J Med Mycol.

[7] Radhakrishnan M, Balaji S, Balagurunathan R (2007). Thermotolerant actinomycetes from the Himalayan Mountains-Antagonistic potential, characterization and identification of selected strains. Malay Appl Biol.

[8] Mayuran S, Radhakrishnan M, Balagurunathan R (2006). Antimicrobial pigments from desert soil actinomycetes. In: proceeding of the National Symposium on “Recent Trends in Microbial Biotechnology”. Sri Sankara Arts and Science College.

[9] Balagurunathan R, Subramanian A (1993). Studies on marine Streptomyces nigrifaciens P-9. Taxonomy and standardization of antibiotic production. Ciencias Marinas.

[10] Balagurunathan R, Subramanian A (2001). Antagonistic streptomycetes from marine sediments. Adv Biosci.

[11] Rahalison L, Hamburger M, Monod M, Frenk E, Hostetmann K (1993). Antifungal tests in phytochemical investigations: Comparison of bioautographic methods using phytopathogenic and human pathogenic fungi. Planta Medica.

[12] Fiedler HP (1993). Biosynthetic capacities of actinomycetes. Screening for secondary metabolites by HPLC and UV-visible absorbance spectral libraries. Nat Prod Lett.

[13] Ilic SB, Kontantinovic SS, Todorovic ZB (2005). UV/Vis Analysis and antimicrobial activity of Streptomyces isolates. Med Biol.

[14] Jensen PR, Mincer TJ, Fenical W (2003). The true potential of the marine micro organism. Curr Drug Discov.

[15] Lachevalier HA (1975). Production of the same antibiotics by members of the different genera of microorganisms. Adv Appl Microbiol.

[16] Okami Y (1986). Marine microorganisms as a source of bioactive agents. Microb Ecol.

[17] Gonzallez JB, Fernandez FJ, Tomasini A (2003). Microbial secondary metabolites production and strain improvement. Indian J Biotechnol.

[18] Sathi ZS, Sugimoto N, Khalil MI, Gafur MA (2002). Isolation of yellowish antibiotic pigment 4-hydroxy nitrobenzene from a strain of Streptomyces. Pak J Biol Sci.

[19] Scorzoni L, Benaducci T, Almeida AM, Silva DH, Bolzani VS, Gianinni MJ (2007). The use of standard methodology for the determination of antifungal activity of natural products against medical yeasts Candida sp and Cryptococcus sp. Braz J Microbiol.

[20] Zahner H, Dahlbohm R, Nilsson JL (1985). Methods in the search for new secondary metabolites from microorganisms, a comparison. VIII International Symposium on Medicinal Chemistry, Proceedings.

[21] Saha S, Thavasi R, Jayalashmi S (2008). Phenazine pigments from Pseudomonas aeruginosa and their application as antibacterial agent and food colourants. Res J Microbiol.

